# Dufourmentel Rhomboid Flap for Plantar Charcot Midfoot Ulcer: A Novel Reconstructive Approach

**DOI:** 10.7759/cureus.81484

**Published:** 2025-03-30

**Authors:** Moamen Elhaddad, Alexander Carrillo-Kashani, Karina Tavakalyan, Behnam David Massaband

**Affiliations:** 1 Foot and Ankle Surgery, Cedars-Sinai Medical Center, Los Angeles, USA

**Keywords:** charcot neuroarthropathy, dufourmentel flap, exostectomy, limb salvage surgery, plantar midfoot ulcer, rhomboid flap, tendo-achilles lengthening

## Abstract

Chronic plantar ulcers in Charcot neuropathic osteoarthropathy (CNO) present a significant challenge in limb salvage due to biomechanical instability, poor tissue quality, and high mechanical stress at weight-bearing sites. Traditional surgical techniques, such as exostectomy and tendo-Achilles lengthening (TAL), effectively redistribute plantar pressure but often fail to provide durable soft tissue coverage. This study describes the first documented use of a Dufourmentel rhomboid flap for reconstructing a chronic plantar midfoot ulcer in a 59-year-old female with CNO and poorly controlled diabetes. The patient presented with a non-healing ulcer over a prominent osseous deformity, complicated by advanced neuropathy and a rocker-bottom foot. Surgical intervention included TAL, exostectomy, and meticulous wound debridement, followed by primary closure using a Dufourmentel flap to achieve tension-free, durable coverage. Postoperatively, the patient was managed with strict immobilization using a total contact cast and transitioned to a Charcot Restraint Orthotic Walker (CROW) boot. Despite partial non-compliance with weight-bearing restrictions, the wound healed completely by six months, with no recurrence. This case highlights the Dufourmentel flap as an innovative and effective reconstructive option for complex plantar ulcers in CNO, offering enhanced soft tissue resilience and long-term stability. The integration of TAL, exostectomy, and biomechanically optimized wound closure provides a comprehensive approach to limb salvage in high-risk diabetic patients. Further research is warranted to evaluate the flap’s long-term outcomes and broader applicability in Charcot foot reconstruction.

## Introduction

Charcot neuropathic osteoarthropathy (CNO) is a progressive, neuropathic disorder characterized by joint dislocation, bone fragmentation, and structural collapse, predominantly affecting the midfoot. The resulting osseous deformities and abnormal pressure distribution frequently lead to chronic ulcerations, posing a significant challenge in limb salvage [1]. Well-established surgical techniques, including exostectomy and tendo-Achilles lengthening (TAL), play a crucial role in redistributing plantar pressure and promoting wound healing in neuropathic patients [1,2]. However, achieving durable soft tissue coverage in these cases remains difficult, particularly in patients with poor tissue quality and high mechanical stress at weight-bearing sites.

Traditional closure techniques often fail to provide adequate, long-term coverage for deep, non-healing plantar ulcers, especially when subcutaneous fat or muscle is exposed. Skin grafts and local advancement flaps have been used, but their durability in high-pressure areas is limited, increasing the risk of wound dehiscence and recurrence [3-5]. The Dufourmentel rhomboid flap, first described in 1962, is a geometric transposition flap designed to optimize tissue redistribution, preserve vascular integrity, and minimize donor site morbidity [6]. This technique offers tension-free closure and enhanced soft tissue resilience, making it a viable reconstructive option for complex wounds. Despite its established success in reconstructive surgery, its application in Charcot foot wounds has not been previously documented.

This case presents the first reported use of a Dufourmentel rhomboid flap for a chronic plantar ulcer in CNO, demonstrating its efficacy in achieving complete wound healing. By integrating TAL, exostectomy, and biomechanically optimized wound closure, we highlight a comprehensive, multidisciplinary approach that not only offloads pressure points but also ensures stable, long-term wound coverage in a high-risk diabetic patient.

## Case presentation

**Figure 1:**
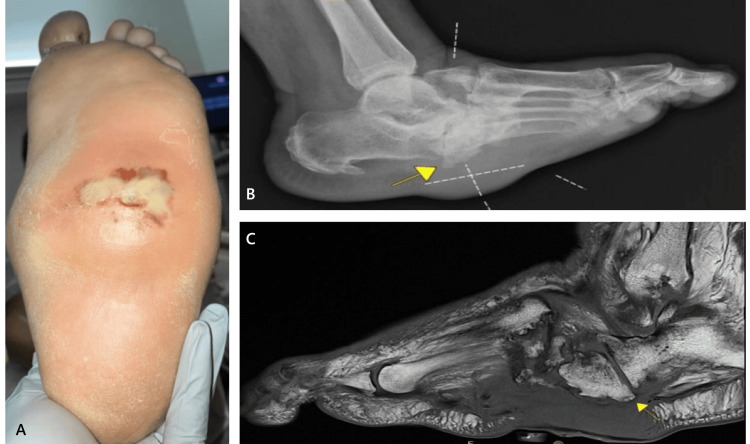
Preoperative clinical and imaging assessment. (A) Preoperative view of the non-healing plantar midfoot ulcer on the left foot, demonstrating a defect with yellow fibrinous exudate and surrounding erythematous tissue. (B) Pre-operative non-weight-bearing lateral radiograph of the left foot demonstrating chronic neuropathic midfoot collapse with a plantar exostosis (arrow) underlying the ulcer site. Associated soft tissue swelling is present. (C) T1-weighted sagittal MRI view of the left foot demonstrating inflammatory fluid accumulation at the plantar midfoot without evidence of progressive osteomyelitis. Additional findings include neuropathic arthropathy with subluxation/dislocation, cortical irregularity at the midfoot joints, and midfoot sag (arrow), consistent with advanced CNO. CNO: Charcot neuropathic osteoarthropathy

A 59-year-old female with a history of hypertension, poorly controlled diabetes mellitus (HbA1c 7.7%), CNO, and prior right hallux amputation presented with a non-healing plantar midfoot ulcer on the left foot (Figure 1, panel A). Over the preceding weeks, she reported progressive swelling and serous drainage from the wound but denied fever, chills, or systemic symptoms.

On examination, a soft, fluctuant mass was palpable beneath the ulcer, initially raising concerns for an underlying abscess or deep infection. The plantar midfoot ulcer measured 3.0 × 2.5 × 1.0 cm, extending to the muscle layer without overt signs of infection; specifically, no erythema, warmth, or purulent drainage was noted. Further assessment revealed that the fluctuance resulted from serous and fibrinous fluid accumulation mixed with subcutaneous fat rather than an abscess.

The left foot demonstrated a rocker-bottom deformity, characteristic of advanced CNO. Pedal pulses were palpable. Neurologically, the patient exhibited loss of protective sensation (as confirmed using the Semmes-Weinstein 10 g monofilament) in the left foot, consistent with advanced diabetic neuropathy. A positive Silfverskiöld test indicated gastrocnemius-soleus equinus, which likely contributed to increased plantar forefoot pressure and the chronicity of the ulcer. Laboratory evaluation revealed mild anemia (Hb: 10.4 g/dL) and elevated C-reactive protein (CRP: 25.6 mg/L), while the white blood cell count remained within normal limits, suggesting chronic inflammation rather than an acute infectious process.

Imaging confirmed severe CNO with midfoot collapse. Plain radiographs of the left foot demonstrated chronic neuropathic changes with plantar exostosis at the wound site (Figure 1, panel B). Vascular ultrasound and ankle-brachial index (ABI) testing were within normal limits (left ABI: 1.0), indicating preserved perfusion.

MRI revealed mild marrow edema within the talus and anterior calcaneus, likely reflecting sequelae of prior reactive marrow changes or early osteomyelitis. Additionally, inflammatory fluid accumulation was noted at the plantar midfoot, without evidence of progressive osteomyelitis. Further findings were consistent with neuropathic arthropathy, including subluxation/dislocation and cortical irregularity at the tarsometatarsal, naviculocuneiform, talonavicular, and calcaneocuboid joints, as well as pes planus and midfoot sag, further confirming the severity of the underlying structural deformity (Figure 1, panel C). Due to the ulcer’s chronic nature and history of drainage, a deep tissue culture was obtained preoperatively, which yielded no evidence of infection.

Given the absence of overt infection and the presence of a chronic non-healing ulcer over a prominent osseous deformity, surgical intervention was pursued. The planned procedure included a TAL, exostectomy for offloading the surgical wound, and wound debridement, with wound closure considered based on intraoperative findings.

During surgery, the wound was carefully examined, revealing serous fibrinous exudate with subcutaneous plantar fat but no purulence, abscess, or signs of deep infection. A bony prominence at the cuboid, confirmed via fluoroscopy, was excised to alleviate plantar pressure, followed by a TAL to improve offloading and reduce forefoot/midfoot plantar pressure. An intraoperative midfoot bone biopsy was taken for pathology and cultures, which later tested negative, confirming the absence of osteomyelitis or soft tissue infection.

Given the clean wound bed, improved pressure distribution following exostectomy, and TAL, the surgical team proceeded with primary closure using a Dufourmentel rhomboid flap, ensuring durable soft tissue coverage and minimizing tension at the closure site.

On postoperative day one, the wound remained intact with no signs of dehiscence or infection, and a total contact cast (TCC) was applied for strict immobilization. Upon discharge, the patient was prescribed oral cephalexin 500 mg four times daily for seven days as prophylaxis. No anticoagulants were administered, as the patient had no history of thromboembolism and was ambulating partially using a walker. The TCC was maintained for two weeks and then changed weekly until week four, when sutures were removed. At that point, the patient transitioned to a Charcot Restraint Orthotic Walker (CROW) boot for ongoing long-term offloading.

**Figure 2:**
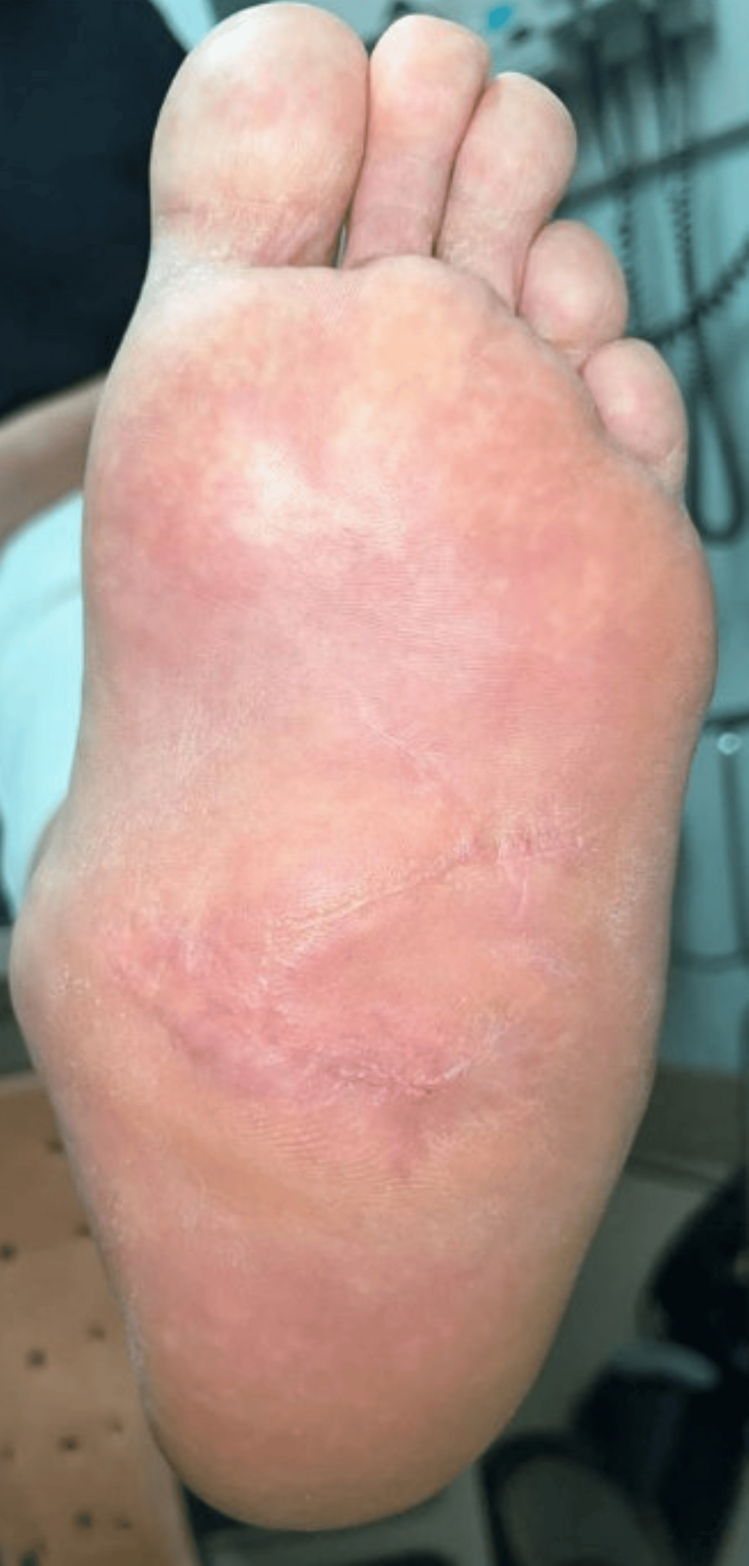
Healed plantar midfoot following Dufourmentel flap reconstruction. Postoperative clinical image demonstrating complete wound healing at six months. The flap remains well-integrated with no signs of dehiscence, infection, or hypertrophic scarring.

Despite strict non-weight-bearing instructions, the patient engaged in partial ambulation, resulting in mild wound maceration and minimal dehiscence. Management included betadine application, strict immobilization, and reinforcement of weight-bearing restrictions, leading to an uneventful progression toward healing. By six months postoperatively, the wound had completely healed (Figure 2). Given the chronic nature of her Charcot deformity, the patient was instructed to remain in a CROW boot indefinitely for long-term pressure redistribution and prevention of recurrent ulceration.

Surgical technique

**Figure 3:**
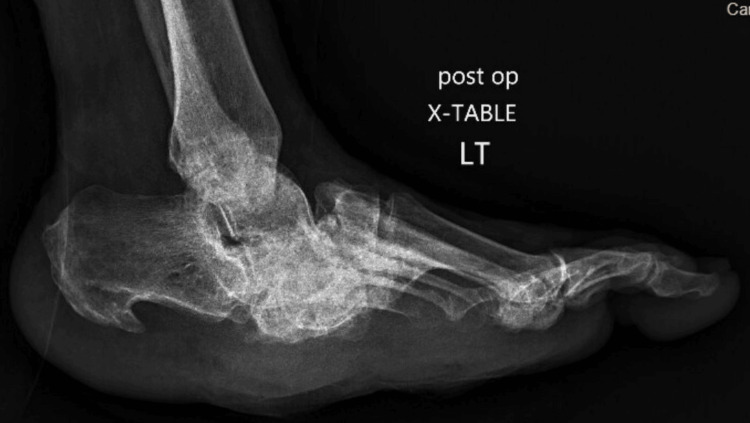
Postoperative lateral radiograph. Immediate postoperative non-weight-bearing lateral radiograph demonstrating resolution of the plantar exostosis after exostectomy, with realignment of the midfoot contour and offloading of the ulcer site.

The patient was positioned supine on a radiolucent table with the leg elevated on a foam wedge. A 10 mm lateral incision was made at the junction of the thick plantar and softer lateral foot skin, followed by meticulous dissection using a soft tissue elevator to create a working portal while protecting vital structures and minimizing thermal injury. With the foot in neutral or dorsiflexion, a 2-5 mm high-torque, low-speed (6000 rpm) burr was used for gradual exostectomy under fluoroscopic guidance to ensure precise bony debridement and offloading of the ulcer site (Figure 3).

To address equinus deformity, a triple-hemisection Achilles tendon lengthening was performed. Small transverse incisions were made at the medial, central, and lateral aspects of the tendon, followed by a stepwise transection of the fibers. Gradual dorsiflexion was applied, achieving 10-15 degrees of dorsiflexion without excessive force to reduce plantar pressure.

The wound was then circumferentially excised using a #15 blade, removing all non-viable and necrotic tissue, resulting in a 4.0 × 4.0 × 1.0 cm defect extending to the muscle layer. Given the absence of infection and the need for durable coverage, a rhomboid Dufourmentel flap was planned and executed.

Dufourmentel flap execution

**Figure 4:**
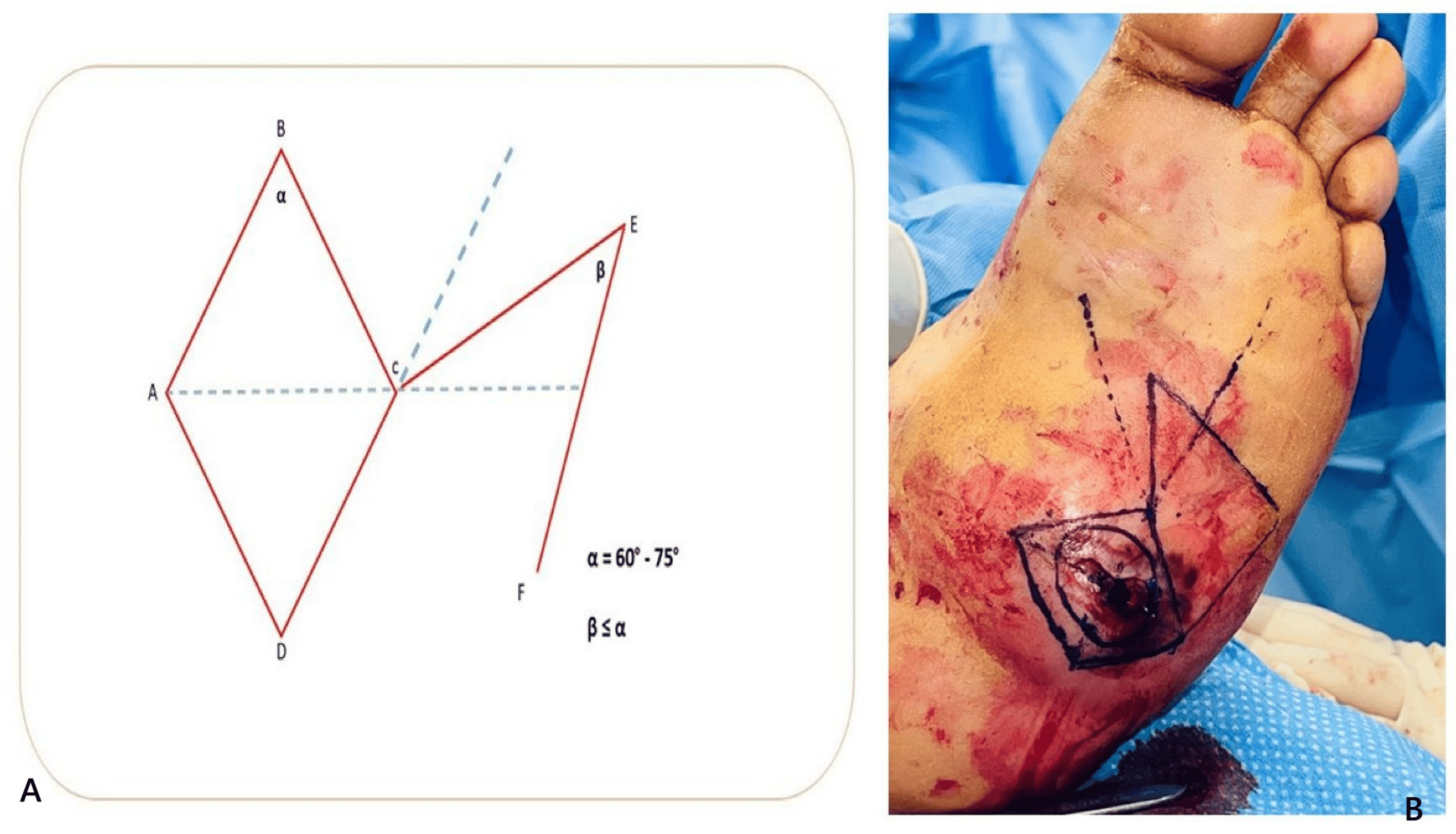
Dufourmentel rhomboid flap design and clinical execution. (A) Illustration showing rhomboid flap planning. Red solid lines indicate surgical incisions for wound excision and flap elevation. Blue dotted lines represent imaginary guides for flap orientation and angle planning. The flap pivot (D-F) was later depicted intraoperatively in the right using a solid marker line. (B) Intraoperative biogeometric skin markings replicating the schematic design, demonstrating translation of the flap plan to clinical application. The illustration is created by the authors of this study.

The Dufourmentel rhomboid flap is designed to achieve tension-free closure while minimizing cutaneous deformity at the pivot point of transposition. Proper geometric planning is essential (Figure 4, panel A), with the acute angle (α) of the defect ranging between 60° and 75° for optimal flap positioning. The first incision bisects the angle between the short diagonal axis of the rhomboid defect and its adjacent side, with its length matching one side of the defect. The flap angle (β) may equal or be slightly smaller than α, allowing for flexibility in design. Unlike the Limberg rhombic flap, which precisely conforms to the defect, the Dufourmentel flap relies on secondary movement of the surrounding skin to facilitate closure, making it well-suited for high-tension areas.

To ensure precise excision and optimal flap transposition, preoperative bio-geometric planning was performed using skin markings (Figure 4, panel B). The flap was sharply dissected to the level of the subcutaneous tissue and fascia, then carefully elevated and transposed into the defect, ensuring proper tissue alignment and vascular perfusion.

**Figure 5:**
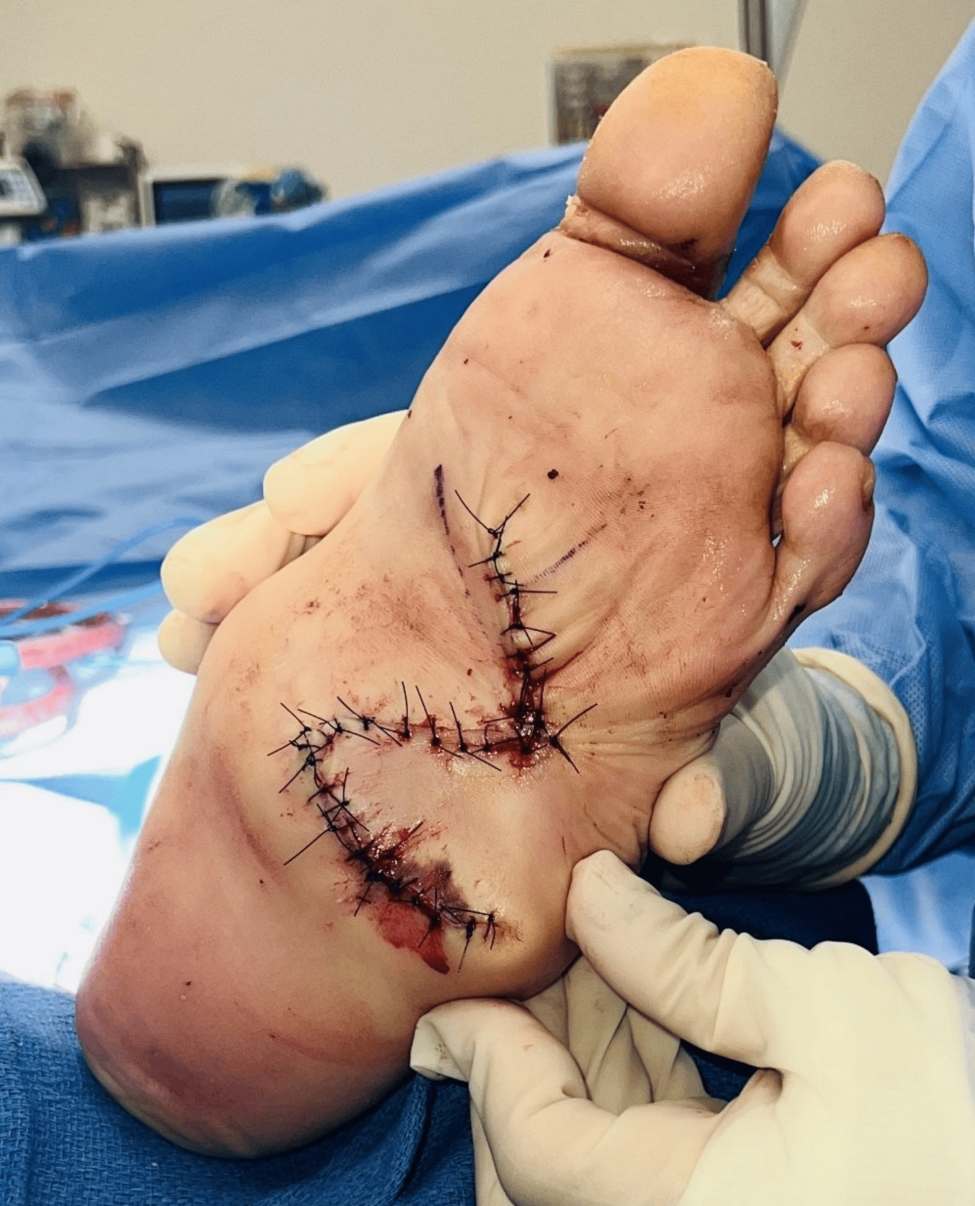
Final intraoperative view following flap transposition and layered closure. The Dufourmentel rhomboid flap is well-positioned with secure approximation, ensuring tension-free adaptation and optimized vascular integrity for durable soft tissue coverage.

The surgical field was thoroughly irrigated with normal saline to minimize contamination. Layered closure was performed using 3-0 absorbable polyglactin 910 sutures (Vicryl) for the subcutaneous layer and 4-0 non-absorbable nylon sutures for the skin, ensuring a secure, tension-free adaptation to the recipient site (Figure 5). A protective wound dressing was applied, followed by controlled ankle motion (CAM) boot placement to enhance stabilization and offloading.

## Discussion

CNO presents a significant challenge in limb preservation, particularly when complicated by chronic plantar ulcerations. The interplay of neuropathy, biomechanical instability, and abnormal pressure distribution often leads to progressive tissue breakdown. While established interventions such as exostectomy and TAL have been widely employed to offload plantar pressure and facilitate ulcer healing, achieving durable soft tissue coverage in high-stress weight-bearing areas remains a surgical challenge [1,2].

Traditional closure methods, such as skin grafting and local advancement flaps, have shown limited durability on the plantar foot due to the high mechanical stress of weight-bearing, often resulting in recurrence and dehiscence [3-5]. Skin grafts, in particular, are generally not recommended for diabetic foot wounds on plantar surfaces because of their poor resistance to shear forces. Local advancement flaps may provide improved outcomes compared to skin grafts and have demonstrated reliable results in selected cases, especially for small, shallow defects with well-vascularized surrounding tissue. However, their effectiveness can still be limited in deeper or larger plantar ulcers due to tension across the closure and the thickness of plantar skin.

Other options for plantar defect reconstruction include fasciocutaneous flaps, particularly the medial plantar artery flap, which provides well-vascularized, durable coverage suitable for weight-bearing regions. However, these flaps require extensive dissection and are associated with donor-site morbidity and sacrifice of the plantar cutaneous nerve [7,8]. Free flaps such as the anterolateral thigh or gracilis muscle flap have also been described for larger or recurrent diabetic foot defects, but they require microsurgical expertise and carry a higher risk of failure in patients with microvascular compromise [9,10]. In comparison, the Dufourmentel rhomboid flap offers a geometrically optimized transposition technique that preserves vascular integrity and minimizes donor-site morbidity, making it a practical and versatile option for localized plantar ulcers with preserved perfusion.

The Dufourmentel rhomboid flap, originally described in 1962, is a local transposition flap that allows tension-free closure while preserving vascular integrity and maintaining the biomechanical properties of the plantar skin [6]. Unlike traditional advancement or rotation flaps, the rhomboid design redistributes tension away from the defect, minimizing wound stress and enhancing long-term stability [11]. In reconstructive surgery, rhomboid flaps have demonstrated high success rates in achieving durable closure in pressure-prone areas like sacral pressure sores, making them a compelling option for Charcot-related plantar defects [12]. To our knowledge, this is the first documented application of a Dufourmentel flap for a Charcot midfoot ulcer, demonstrating its effectiveness in achieving complete epithelialization without recurrence at six months.

The vascular supply of the Dufourmentel flap is derived primarily from the dermal-subdermal plexus, which is typically preserved during elevation and supports flap reliability in well-perfused tissue. In diabetic patients, microvascular compromise can limit flap viability due to reduced capillary perfusion, increasing the risk of delayed healing or necrosis [13]. However, emerging evidence suggests that patients with Charcot neuroarthropathy may retain relatively better peripheral cutaneous microvascular reactivity compared to those with uncomplicated diabetic neuropathy, potentially improving flap outcomes in this subgroup. This was particularly relevant in the present case, where the patient had Charcot foot despite underlying diabetes and demonstrated adequate wound healing without vascular complications [14].

TAL is well-documented in the literature as a crucial adjunct for reducing forefoot pressure and enhancing ulcer healing in patients with diabetic neuropathy and equinus contracture [15]. By increasing ankle dorsiflexion, TAL redistributes weight posteriorly, shifting load from the midfoot and forefoot to the hindfoot, thereby lowering peak plantar pressures at the ulcer site, improving healing rates, and reducing recurrence [16-18]. Roger et al. reported that combining TAL with TCC may further mitigate midfoot deforming forces and reduce Charcot-related morbidity [1].

Exostectomy, another key component of this surgical approach, directly targets osseous prominences that serve as pressure points, particularly in Charcot deformities where midfoot collapse leads to excessive plantar bony contact [19]. Literature supports exostectomy as an effective limb-salvage procedure for neuropathic ulcers in cases of stable deformity, with reported ulcer healing rates of approximately 75% following the intervention [20]. Moreover, when paired with accommodative bracing, exostectomy appears to yield more favorable outcomes in patients without active ulceration [19,21,22].

Despite achieving successful initial closure, neuropathic patients remain at high risk for re-ulceration due to persistent biomechanical instability and impaired protective sensation. Strict adherence to offloading protocols is crucial, as premature weight-bearing is a leading cause of wound dehiscence and breakdown in Charcot patients [23,24-26]. Literature strongly supports the use of TCC to ensure effective pressure redistribution and minimize ulcer recurrence [23-25]. In this case, the combination of TAL and exostectomy, followed by primary closure with a Dufourmentel flap and staged immobilization with TCC and CROW boot, provided an optimal strategy for wound healing and long-term structural stability.

## Conclusions

Chronic plantar ulcers in CNO pose a significant surgical challenge due to biomechanical instability and poor soft tissue quality. This case demonstrates the first reported use of a Dufourmentel rhomboid flap for Charcot midfoot ulcer reconstruction, achieving complete healing and durable coverage when combined with exostectomy and TAL. The flap’s tension redistribution and vascular preservation make it a promising option for weight-bearing wounds.

Further research is needed to assess its long-term efficacy and comparative outcomes in Charcot foot reconstruction, guiding future strategies for limb salvage and ulcer prevention in high-risk diabetic patients.
